# Statistical Morphology and Fragment Mapping of Complex Proximal Humeral Fractures

**DOI:** 10.3390/medicina59020370

**Published:** 2023-02-14

**Authors:** Karen Mys, Luke Visscher, Kenneth Petrus van Knegsel, Dominic Gehweiler, Torsten Pastor, Amirsiavosh Bashardoust, Anna Sophie Knill, Carolin Danker, Jan Dauwe, Rayna Mechkarska, Georgi Raykov, Grzegorz Marek Karwacki, Matthias Knobe, Boyko Gueorguiev, Markus Windolf, Simon Lambert, Stefaan Nijs, Peter Varga

**Affiliations:** 1 AO Research Institute Davos, 7270 Davos, Switzerland; 2 Royal Brisbane and Women’s Hospital, 4029 Brisbane, Australia; 3 Centre for Biomedical Technologies, Queensland University of Technology, 4000 Brisbane, Australia; 4 Department of Orthopedic and Trauma Surgery, Lucerne Cantonal Hospital, 6000 Luzerne, Switzerland; 5 Department of Trauma Surgery, University Hospitals Leuven, 3000 Leuven, Belgium; 6 University Multiprofile Hospital for Active Treatment and Emergency Medicine “N. I. Pirogov”, 1606 Sofia, Bulgaria; 7 Medical University of Varna ‘‘Prof. Dr. Paraskev Stoyanov’’, 9002 Varna, Bulgaria; 8 Department of Radiology and Nuclear Medicine, Lucerne Cantonal Hospital, 6000 Luzerne, Switzerland; 9 University College London Hospital, London NW1 2BU, UK; 10 University Medical Center Utrecht, 3584 CX Utrecht, The Netherlands

**Keywords:** proximal humerus fracture, computed tomography, fracture morphology, fragment, comminution, probability map, statistical model

## Abstract

*Background and Objectives*: Proximal humerus fractures (PHFs) are common in the elderly, but the treatment results are often poor. A clear understanding of fracture morphology and distribution of cortical bone loss is important for improved surgical decision making, operative considerations, and new implant designs. The aim of this study was to develop a 3D segmentation fracture mapping technique to create a statistical description of the spatial pattern and cortical bone loss of complex PHFs. *Materials and Methods*: Fifty clinical computed tomography (CT) scans of complex PHFs and their contralateral intact shoulders were collected. In-house software was developed for semi-automated segmentation and fracture line detection and was combined with manual fracture reduction to the contralateral template in a commercial software. A statistical mean model of these cases was built and used to describe probability maps of the fracture lines and cortical fragments. *Results*: The fracture lines predominantly passed through the surgical neck and between the tuberosities and tendon insertions. The superior aspects of the tuberosities were constant fragments where comminution was less likely. Some fracture lines passed through the bicipital sulcus, but predominantly at its edges and curving around the tuberosities proximally and distally. *Conclusions*: A comprehensive and systematic approach was developed for processing clinical CT images of complex fractures into fracture morphology and fragment probability maps and applied on PHFs. This information creates an important basis for better understanding of fracture morphology that could be utilized in future studies for surgical training and implant design.

## 1. Introduction

Proximal humerus fractures (PHFs) are the third most common fractures in the population over 65 years of age. PHFs represent approximately five percent of all fractures and continue to become more frequent as the prevalence of osteoporotic fractures increases due to increased aging [1]. The incidence of PHFs is two to three times higher in women compared with men [2]. The majority of PHFs are treated non-operatively but, where complexity and displacement are considered sufficient to predicate a poor outcome after healing, operative intervention has been considered to provide improved outcomes, although this remains contentious. The primary options for the operative treatment of PHFs in the elderly are open reduction and internal fixation (ORIF) with angular fixed plating, and reverse total shoulder replacement [3]. As a general principle in fracture surgery, preserving the patients’ native bone is preferred. However, plate fixation of PHFs has high reported technical failure rates, with loss of fracture reduction, secondary loss of fragment position, and screw penetration through the articular as the major complications [4,5,6,7].

PHFs are often complex fractures composed of four major segments: the lesser tuberosity, greater tuberosity, head, and shaft. Moreover, the fragments are often displaced and maintained by the multidirectional pull of the rotator cuff insertions and capsular attachment [8,9]. Additionally, high energy transfer through fragile (osteopaenic and -porotic) bones frequently results in displacement-specific zones of comminution that are correlated with fracture instability and delayed or non-union. Impaction can result in interfragmental bone loss in the toroidal region between the head and the shaft, which is an important anatomic and functional area [10,11]. The most important predictive factor for a successful outcome after angular plate screw osteosynthesis of PHFs is the maintenance of an optimal (anatomic) reduction of the fracture [4]. A prerequisite for reducing the fracture fragments to the anatomical premorbid status is an accurate comprehension of the fracture pattern. Therefore, understanding the entire three-dimensional (3D) extent of the fracture is an essential step in preoperative planning. A preoperative radiograph or 2D slice-wise view of computed tomography (CT) scan may not be sufficient for a complete comprehension of complex PHF. The added value of advanced visualization of segmented 3D CT images is positively associated with a better ability of both less and more experienced surgeons to categorize complex fractures [12,13,14]. Having appreciated the complexity of the PHF, the subsequent challenge is to define the premorbid intact anatomic morphology so that accurate reduction tactics and endpoints can be planned and achieved. An improved understanding of fracture morphology and corresponding zones of comminution may therefore be the foundation for all aspects of surgical care, including decision making between treatment options, preoperative planning, surgical approach, and reduction and fixation strategies, as well as optimizing implant design.

Fracture mapping was pioneered by Armitage et al. in the analysis of fracture morphology in operatively treated scapular fractures [15]. They proposed a two-dimensional (2D) manual fracture reduction on a 2D template. A similar fracture mapping approach was applied on the proximal humerus by Hasan et al. [16]. However, the manual translation of fracture lines in 2D images has the potential to misrepresent the true anatomical condition by observer bias and error and by underestimating complex anatomical variance: this problem may be addressed by using 3D analyses. As an example, Dugarte et al. showed that a 3D analysis of scapular fracture morphology was more accurate than the 2D method [17]. This technique has been adapted and applied to several anatomical locations [16,18,19,20,21,22,23]. Most studies defined a single fracture line transcribed from the position between two fragments. However, in osteoporotic proximal humerus fractures, there is commonly a loss of definition of the edge of the cortical bone fragments on cross-sectional imaging. Comminution zones have been investigated in pilon fractures [24]. However, this was performed using a single slice technique, and there has been little investigation into the representation of 3D cortical fragment and bone loss, both of which are important for the assessment of likely intrinsic fracture stability after attempted reduction. The 3D morphology of PHF has recently been reported with a focus on greater tuberosity fragmentation [25], but the comminution was not analyzed, despite the influence of the latter on surgical outcomes [10].

The aim of this study was to develop a 3D segmentation fracture-mapping technique to create a statistical description of the morphology and cortical bone loss of complex multifragmentary PHFs.

## 2. Materials and Methods

**Figure 1:**
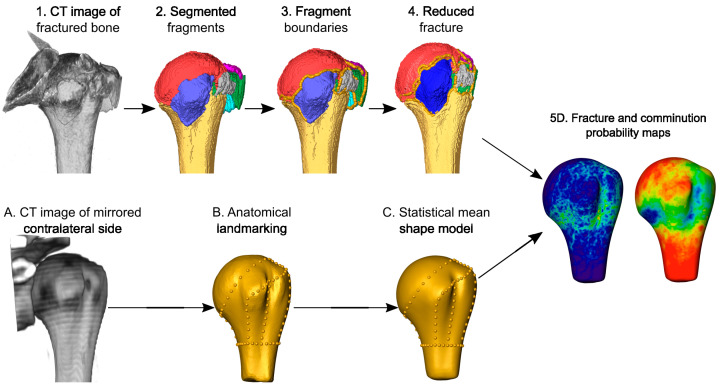
Overview of the study methodology. The cortical fragments of the fractured proximal humerus were segmented on the CT image (1, 2), the fragment boundaries were determined (3), and the fracture was virtually reduced (4). The contralateral intact side was mirrored (A), landmarked at key anatomical features (B), and a statistical mean shape model was calculated from the data of all cases (C). The fracture lines and fragment areas were projected onto the statistical shape model and superimposed for all cases, providing spatial statistical descriptions of the fracture morphology and fragments in the form of probability maps (5D).

Fifty computer tomography (CT) scans of complex multifragmentary proximal humerus fractures were retrospectively collected, semi-automatically segmented, and virtually reduced. A statistical shape model of the intact proximal humerus was generated from the contralateral bones and the fracture lines and fragments were systematically mapped onto the mean model to provide probability maps of the fracture morphology and the fragments, respectively (Figure 1).

### 2.1. Data Collection

CT scans of patients with three- and four-part proximal humerus fractures and including the intact contralateral side were retrospectively collected at the Lucerne Cantonal Hospital. The study was approved by the local Ethical Committee (Nr. 2021-00823). The patient database was searched between 2009 and 2020 for appropriate cases. The inclusion criteria were age >18 years, radiologically confirmed closed fracture of the proximal humerus, displaced or unstable 3- or 4-part fractures of the proximal humerus, intact contralateral humerus, and that the contralateral humerus was present on the clinical overview CT reconstruction. The exclusion criteria were pathological fractures, re-fractures, contralateral proximal humerus fracture, the presence of an implant or prosthesis, and inadequate resolution of the CT reconstruction.

The data of fifty patients (40 women, 10 men, x left, y right) with ages ranging from 27 to 89 years (mean ± standard deviation (SD): 68.5 ± 13.1 years) were selected for further analysis. The CT images were acquired using various scanners according to the standard of care of the study site (120 kVp, 140 mAs, and 0.8 pitch). The reconstructions included (1) an overview image with a slice thickness of 2.3 mm, 512 × 512 matrix, mean pixel size of 0.84 mm, and “I30f” kernel and (2) a zoom-in image of the ipsilateral fractured shoulder with a mean slice thickness of 0.55 mm, 512 × 512 matrix, mean pixel size of 0.42 mm, and “I30f” kernel.

### 2.2. Fracture Fragment Segmentation

**Figure 2:**
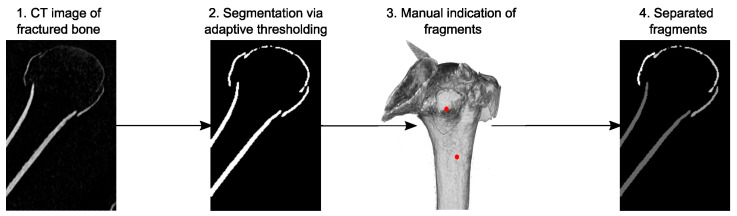
Semi-automatic segmentation of fracture fragments. The CT image (1) was automatically segmented utilizing an adaptive thresholding approach (2), remaining connected fragments were isolated with minimal user interaction to indicate the parts to be separated (3, red points). Finally, the fragments were separated automatically based on the grayscale gradients of the CT image (4).

The cortical compartments of the fracture fragments were identified and isolated on the zoom-in CT images using a custom image processing tool and graphical user interface (GUI) implemented in Matlab R2021b (The Mathworks Ltd., Natick, MA, USA) and C++, established in a previous study [12]. The procedure automatically pre-segmented the cortical shell utilizing an adaptive thresholding technique [26] combined with an edge-detection segmentation algorithm. The tool recognized the detached fragments and enabled the separation of the not fully detached parts semi-automatically with minimal user interaction (Figure 2). This was achieved by calculating the gradients for each fragment and removing the voxel with the highest gradient until the pre-selected fragments were no longer connected. Subsequently, all the voxels that did not reconnect were added back to the fragments. Manual corrections were finally performed in Amira 3D 2012.2 (Thermo Fisher Scientific, Waltham, MA, USA) to capture the fine details for the detached fragments. This workflow enabled efficient processing of the large number of CT data, which would have taken too long with standard tools and manual work.

### 2.3. Fracture Line Detection

**Figure 3:**
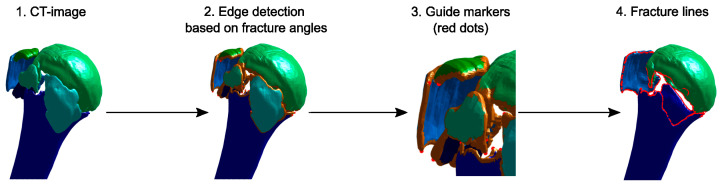
Semi-automatic detection of fracture lines on the segmented CT scans (1). For each fragment, high curvature areas were identified (2, thick brown areas) and guide markers were proposed to indicate the fracture path (3, red dots), which could be manually adapted to improve the line. Finally, the fracture lines were calculated by optimizing a cost–function based on the distance to the next free boundary and the fracture angle (4, red lines; note that some fragments were not shown here for better visualization).

The boundary line of each fracture fragment was identified semi-automatically (Figure 3) and used to represent the fracture lines. A triangulated surface of each fragment was created from the previously segmented CT images. The algorithm then identified the points on the fragment borders based on the curvature of the surface model. The suggested points could be manually adjusted in the GUI by indicating extra points where the true fracture line should pass through or by deleting erroneous points. Based on this information, the fracture line was calculated as a sequence of spatial points. A more extensive description of this procedure can be found in Appendix A.

### 2.4. Virtual Fracture Reduction

**Figure 4:**
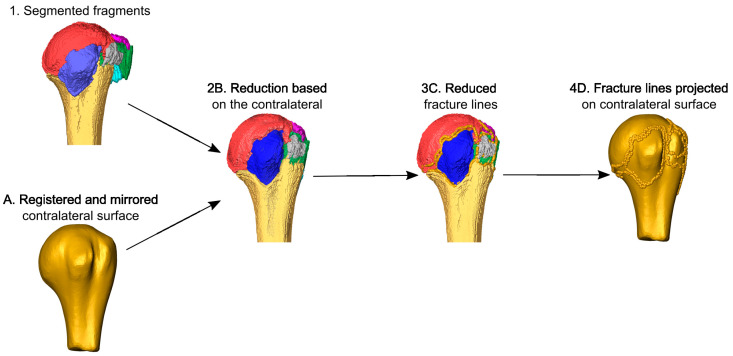
Virtual fracture reduction aided by the intact contralateral side. The fragments of the ipsilateral fracture, indicated by the various colors, (1) were reduced manually based on the surface of the mirrored contralateral bone (A) used as guide (2B). The fracture lines of the reduced fragments (3C) were projected on the contralateral surface (4D).

The fracture was virtually reduced by a surgeon in Amira by solving the 3D-puzzle based on the patient-specific template (Figure 4). All fragments were visualized as surfaces and transformed to be aligned with the mirrored contralateral bone, the surface model of which was superimposed on the ipsilateral side to guide the reduction. The fracture lines of each reduced fragment were automatically projected on the mirrored contralateral surface via closest point matching.

### 2.5. Statistical Fracture Analysis

**Figure 5:**
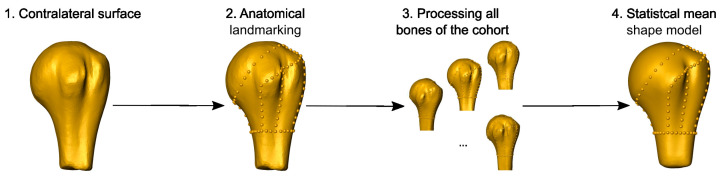
Statistical model of the proximal humerus. Surface models of the intact contralateral bones (1) were labelled manually with anatomical landmarks (2). Data of all cases (3) were used to generate the mean shape model (4).

The fracture lines of all cases were mapped on the mean shape model of the cohort. The latter was created from the intact contralateral proximal humeri. All right bones were mirrored to mimic the left side. Anatomical landmarks were manually identified and marked in Amira and the connecting lines, including secondary landmarks, were automatically calculated. Procrustes analysis was used to align the surfaces of the different bones, resulting in a statistical mean shape model (Figure 5) [27,28].

**Figure 6:**
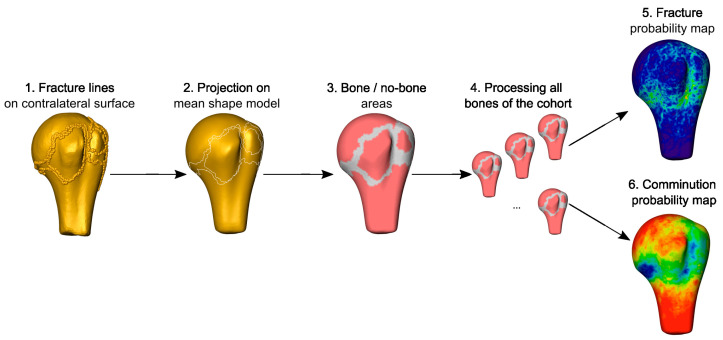
Statistical description of fracture lines and the bone/comminution zones. Fracture lines were projected on the mirrored contralateral surface (1) and mapped onto the mean shape model based on homology (2). Superimposing the results of all cases provided the statistical description of fracture morphology, indicating the probability of fracture lines for each point throughout the surface (5). Similarly, the bony areas were assessed (3) and their probability map was created, indirectly indicating comminution zones (6).

The fracture lines of all cases were then mapped onto the mean shape model based on the corresponding homologies between the individual models and the mean surface. This resulted in the fracture probability map, indicating the frequency of fracture lines passing through the points of the mean surface, i.e., fracture morphology. In a second analysis, the areas of the fragments were projected onto the mean shape, providing the probability maps of cortical bone fragments (Figure 6).

### 2.6. Inter-Operator Reproducibility of the Reduction

The reproducibility of virtual fracture reduction was evaluated based on the fracture lines of 20 bones analyzed by two surgeons. The closest distances of each point of the projected fracture lines on the mean shape model were calculated between the reductions achieved by the two operators and found to be 1.50 ± 1.02 mm (mean ± SD).

## 3. Results

**Figure 7:**
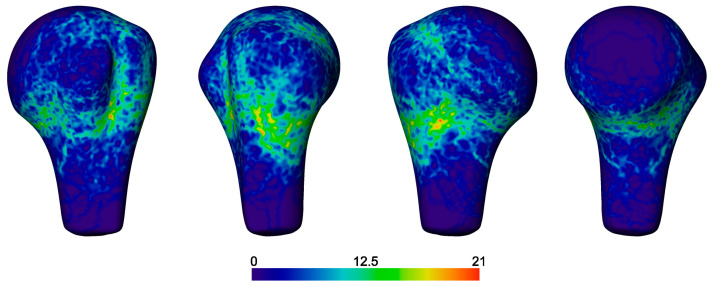
Fracture probability map calculated for all 50 cases. Left to right: Anterior, lateral, posterior, and medial views. The color scale indicates the number of cases having fracture lines passing through the points of the surface map.

The fracture probability map demonstrated a wide distribution of lines that predominated at the surgical neck (Figure 7). There were definite occurrences of fractures along the anatomical neck, but with a lower rate. The area between the surgical and anatomical neck regions likely represents the capsular attachment with a lower fracture frequency. The fracture lines with higher incidences traversed between the known landmarks, i.e., the posterior segment (including most of, but not all, the greater trochanter, GT) and the anterior segment (including the lesser trochanter, LT, and much of the sulcus bicipitalis), and the musculotendinous (capsular) insertion sites on the posterior segment.

Articular fractures were infrequent, with only a few fracture lines splitting the head; most cases showed fracture lines to be clustered at the peripheral <20% of the articular surface. Extension into the proximal humeral shaft occurred, but rarely 3 cm below the surgical neck.

Fracture lines were not frequent within the bicipital sulcus, but were more common at its inferior aspect and at the lateral edge of the sulcus within the posterior segment rather than through the base of the sulcus. The fracture lines frequently followed the curved surface topography of the tuberosities proximally and distally.

**Figure 8:**
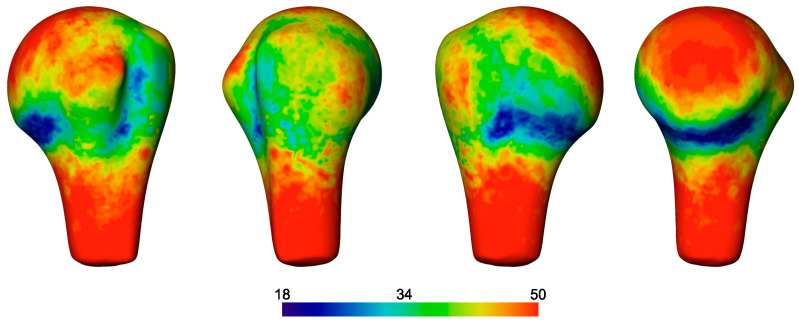
Fragment probability map calculated for all 50 cases. Left to right: Anterior, lateral, posterior, and medial views. The color scale indicates the number of cases having bone available at the points of the surface map.

The fragment probability map demonstrated high rates of comminution at the surgical neck anteriorly (Figure 8). The articular surface of the head and shaft were generally preserved, as were the tendon insertions of the rotator cuff. A high probability of fragmentation was observed posteriorly within the insertion footprint of the capsule, infraspinatus, and teres minor, with more frequent cortical bone fragmentation between the insertions of the latter two muscles, defining these bony regions as separate fragment ‘islands’.

## 4. Discussion

This study presented a systematic CT image processing workflow to generate statistical fracture morphology and fragment probability maps from clinical CT scans and applied it to complex multifragmentary proximal humerus fractures. This was achieved with a custom-developed segmentation tool, computer-assisted semi-automated fracture line detection, and template-aided manual reduction of fracture fragments. A systematic and objective method was utilized to automatically perform anatomy-based mapping of fracture lines to the mean shape model. Moreover, beyond the standard fracture line probability map, we introduced the fragment area probability map that carries important clinically relevant information.

The interest in fracture mapping has recently been increasing with applications for various anatomical locations [16,18,19,20,21,22,23]. The previously presented methods have demonstrated an increasing trend in terms of accuracy. The original 2D mapping techniques were shown to introduce potential inaccuracies and do not support the appreciation of the complex spatial distribution of fracture lines [17]. Dugarte et al. found that half the cases of scapular fractures had fracture lines only visible on 3D analysis and additionally demonstrated deviations in fracture line positions by 10–28 mm: this variation has a profound and potentially clinically significant influence when planning the surgical reduction and fixation tactic. The 2D mapping of fracture lines onto a template also fails to account for the complex 3D anatomical variation that is especially important for the proximal humerus where multiple protruding landmarks and associated tendon insertions exist. Our approach used a 3D-to-3D-mapping technique based on anatomical correspondence through a patient-specific contralateral template and homologous landmark-based matching for the statistical mean shape model that minimizes potential mapping errors that may occur in more simple closest point-based matching approaches. Template-based mapping has been widely used in previous studies, but the level of details can affect the outcomes. Ni et al. acknowledged mismatches in the mapping of calcaneal fractures when using a standard 3D template that did not account for anatomical variability [29]. The use of general templates limits the accountable shape variation to scaling in 3D. Population-wide studies of anatomical bone shape, including in the proximal humerus, demonstrated complex multivariate shape variation in different principal components [27]. To accommodate this variance, the fully automated workflow described here allowed for accurate, reliable, and automated generation of fracture morphology and fragment probability maps.

In the present study, the mirrored intact contralateral side was used as template for fracture reduction that represent the best possible match when the pre-injury status of the ipsilateral site is not available. The influence of the operator on the reduction technique was found to be 1.50 ± 1.02 mm, measured on the fracture lines projected on the mean shape model. Larger differences were observed for small fragments: this was expected since, due to often unclear landmarks and comminution, the fragments did not perfectly fit together, thus rendering the accuracy of the reduction more subjective.

The fragment edges were defined in this study by considering the boundary lines of each separate fragment, in contrast to most other studies transcribing a central line between the fragments. This enabled discrimination between zones of bone fragments and voids, allowing for the definition of bone loss and comminution regions in an automated way. The resulting fragment probability plots demonstrated areas of least fractured regions with seldom damaged bone stock and commonly comminuted zones, resembling ‘tectonic plates’ and ‘water’ maps. The importance of comminution zones relies on their added value compared to the fracture line probability map. Only a few previous studies presented comminution, but those analyses were performed manually [20,24].

A previous study by Hasan et al. investigated fracture morphology in the proximal humerus and provided insight into the morphology of these fractures [16]. Though they used a 2D fracture line mapping method, they confirmed good inter-rater reliability and, for simplicity, represented comminution as single lines. They performed a comprehensive anatomical assessment of fracture lines in relation to osseous zones and based on different 2D views. Most of the findings were in line with our results, with a high frequency around the surgical neck and between tendon insertions that they mapped based on anatomical descriptions. However, division and separate consideration of separate 2D views can lead to some confusion. They assessed a superior view of the fracture map to view the tuberosities form above and generated a frequency graph showing no fractures to pass through the bicipital sulcus on this view. This contrasted with the lateral images in their study that demonstrated vertical fracture lines through the bicipital sulcus. In our study, the fracture lines were frequent through the bicipital sulcus. Ju et al. recently published a 3D fracture morphology study and reported fracture lines through the bicipital sulcus [25]. However, their analysis focused only on fractures around the greater trochanter and relied on a coarser CT image resolution compared to the current study. Further analysis of the fracture morphology map indicated that fractures commonly curved proximally and distally around the tuberosities in Y and inverted Y distributions. This may explain why fractures were described as never passing through the sulcus when assessed from a superior view only. The fractures were more common at either side of the base of the sulcus, breaking off with one tuberosity or the other, or rarely as a separate fragment to both, i.e., the so-called ‘shield fragment’ [30].

The novel fragment probability map demonstrated high rates of comminution at the surgical neck anteriorly. This suggested high rates of comminution due to varus and/or valgus displacement of the head. Hasan et al. reported a high rate of fractures between the supraspinatus and infraspinatus insertions. A similar frequency could be seen in our fracture probability map; however, interestingly, this did not translate to a higher frequency of cortical bone loss in the fragment probability map, which may represent less displacement. The fractures were highly likely to be at the estimated insertions of the capsule, the infraspinatus, and teres minor, and there was more frequent cortical bone loss between the insertions of the latter two muscles, defining them as separate islands.

The results of this study provide clinicians with a more detailed insight into proximal humerus fracture morphology. This may support education regarding the characterization of fragmental marginal comminution and interfragmental stability that can be used to guide a reliable and accurate reduction tactic. The presented information may also contribute to a more accurate fracture classification. Moreover, the results can support implant development. All these aspects were beyond the scope of this work but could be investigated in future studies.

There were technical and methodological limitations to this study. To virtually reduce the fracture fragments correctly, accurate segmentation is essential. However, because of the high complexity of these fractures and the limited resolution of clinical CT scans, small fragments can be and were sometimes missed. Although a previously developed tool [12] was utilized here to assist this procedure, the segmentation technique is also influenced by the skill of the observer. Future work should evaluate the accuracy of clinical CT image-based fracture fragment segmentation against reliable ground truth data, e.g., based on high-resolution (micro-)CT imaging in an in vitro study. Secondly, the quality and integrity of the humeral trabecular (cancellous) bone also has an influence on the stability of the reduction. Consequently, for instance, a large trabecular void negatively impacts the stability. However, with current techniques, it is not possible to identify and separate the trabecular bone accurately on a conventional clinical CT scan due to the limited spatial resolution. Thirdly, the sample size was too small to divide the bones according to their fracture classification. Fourthly, the requirement for a CT scan of the contralateral side is a limitation of the present study as it is not always available clinically; future work could potentially use a predictive shape model for this purpose. This has yet to be validated for fracture reduction, but other work has shown that a predictive shape model can potentially represent the proximal humeral anatomy more accurately than the contralateral side [31].

## 5. Conclusions

This is the first study to achieve systematic and repeatable mapping and statistical analysis of fracture line morphology and bone fragment domains in 3D that accounts for the complex 3D anatomical shape variation in the proximal humerus. The results provided important insights that could be utilized in future studies for surgical education and implant design.

## Data Availability

Not applicable.

## Appendix A

The fracture line was calculated in a two-step algorithm on the meshed fracture, which is partially based on the method developed for archeological fragment reduction by ElNaghy et al. [32]. First, the fracture edge was determined for each fragment. In the second step, a point line was extracted.

In the first step, all triangles $T_{i}\;$that were part of the given fragment edge were identified, by selecting all triangles with a fracture angle $\alpha \left(T_{i}\right)$ higher as a set threshold. To quantify the fracture angle $\alpha \left(T_{i}\right)$, the local neighborhood$\;L\left(T_{i}\right)$ was separated into two groups by a plane along the minimum bending direction v_min_ and the normal vector v_norm_. The fracture angle was defined as the angle between the best fitting planes through each of both groups, considering also the concavity or convexity [32]. To ensure the entire fracture edge was detected, a closing operation was performed.

In the second step, a single point thin line was extracted from the fracture edge. The algorithm proposed guide markers and the user had the option to manually add or remove markers in the GUI. A weight$\;w\left(T_{i}\right)$ was assigned to each triangle $T_{i}$ of the edge E ($T_{i}\in E$) based on the distance to the next free boundary $b\left(T_{i}\right)$ and the fracture angle $\alpha \left(T_{i}\right)$:

$$w\left(T_{i}\right)=b_{i,norm}+\alpha_{i,norm}=\frac{b_{max}-1-b\left(T_{i}\right)}{b_{max}}+\frac{\alpha \left(T_{i}\right)}{\alpha_{max}}$$

The fracture angle $\alpha \left(T_{i}\right)\;$and the boundary distance $b\left(T_{i}\right)$ were each normed to the maximum value $b_{max}:=max\left(b_{i,norm}|i\in m\right)$ and $\alpha_{max}:=max\left(\alpha_{i,norm}|i\in m\right)$, respectively. The center point $C_{i}$ of a triangle $T_{i}$ was selected as a guide marker if it had the lowest weight in its neighborhood $L\left(T_{i}\right)$. Finally, the guide markers were connected by defining the path with the lowest weight between two adjacent markers. This was achieved by creating a graph of the fragment edge where every node represented a triangle of the mesh, and every edge represented the connection of two triangles connected by at least one vertex. For each edge between the two connected triangles $T_{i}$ and $T_{j}$, a cost W(i,j) was determined as:

$$W\left(i,j\right)=\frac{b_{i,norm}+b_{j,norm}}{4}\cdot C_{i}+C_{j}$$
